# Usage activity, perceived usefulness, and satisfaction in a web-based acceptance and commitment therapy program among Finnish ninth-grade adolescents

**DOI:** 10.1016/j.invent.2021.100421

**Published:** 2021-06-24

**Authors:** Tetta Hämäläinen, Kirsikka Kaipainen, Päivi Lappalainen, Anne Puolakanaho, Katariina Keinonen, Raimo Lappalainen, Noona Kiuru

**Affiliations:** a Department of Psychology, Faculty of Education and Psychology, University of Jyväskylä, Finland; b Faculty of Information Technology and Communication Sciences, Tampere University, Finland

**Keywords:** Usage activity, Perceived usefulness, Intervention satisfaction, Acceptance and commitment therapy, Web-based intervention, Adolescents

## Abstract

Understanding adolescent usage activity and experiences in web-based psychological intervention programs helps in developing universal programs that can be adopted for promotion of adolescent well-being and prevention of mental health problems. This study examined the usage activity, perceived usefulness (i.e., learning of mindfulness, acceptance and value-related skills), and program satisfaction of 157 Finnish ninth-grade adolescents, who participated in a school-based five-week universal acceptance and commitment therapy web intervention called Youth Compass. Individual and growth environment-related antecedents were measured before the five-week intervention, adolescents' usage activity during the intervention, and perceived usefulness and satisfaction after the intervention. The results showed that female adolescents and adolescents with high self-regulation were more active program users and had more positive experiences of the program. Most of the adolescents used the program on at least a moderate level and perceived it to be moderately or highly useful and satisfactory. Four subgroups of adolescents were identified based on their usage activity, perceived usefulness, and satisfaction: adolescents in the satisfied group (41%) had average activity and high perceived usefulness and intervention satisfaction, the dissatisfied group (18%) had low activity and very low perceived usefulness and intervention satisfaction, the active group (8%) had very high activity and average perceived usefulness and intervention satisfaction, and the moderate group (33%) had average activity, perceived usefulness and intervention satisfaction. Gender, academic achievement, closeness to mother and teacher, and conflict with teacher were significantly related to subgroup membership. The results suggested that adolescent usage activity, perceived usefulness, and satisfaction with the Youth Compass program may to some extent be predicted based on different factors.

## Highlights

• Most of the adolescents (82%) were moderately to highly satisfied with Youth Compass.
• Program usage and experiences were connected mainly to gender and self-regulation.
• Four user profiles were identified: satisfied, dissatisfied, active, and moderate.
• Profile membership was associated with various individual and environmental factors.

## 1 Introduction

Adolescence begins in puberty and is usually defined to last from around age 12 to 22 (Aalto-Setälä, 2010; Csikszentmihalyi, 2019). It is a rapid developmental phase characterized by a variety of changes such as physical growth, sexual maturation, building autonomy, structural changes in social relations, and adopting new areas of responsibility (Aalto-Setälä, 2010; Lehtonen, 2010). Half of adult mental health problems have their onset before or during adolescence, and disorders related to depression, anxiety, behavior, and substance use represent the most common ones among adolescents (WHO, 2018). The prevalence of mental disorders reach their peak in early adulthood, and it has been estimated that around one in five young people show symptoms that are on a disorder level (Aalto-Setälä, 2010; Marttunen and Kaltiala-Heino, 2007). Therefore, the prevention of mental disorders is a public health priority, and a substantial base of evidence states that effective prevention can strengthen protecting factors and reduce risk factors relating to mental health (WHO, 2004). Early intervention may have long-lasting benefits and prevent the onset and further development of mental health problems. School-based universal preventive intervention programs, i.e., programs delivered to all students instead of selected group, are optimal in wide reach of young people and schools play an important role in adolescent mental health promotion (Mackenzie and Williams, 2018; O'Connor et al., 2018).

Web-based psychological intervention programs have been a growing interest in research due to their accessibility, cost-effectively achievable benefits, and reduced stigma, and have been suggested to be as effective as other treatment formats (Andersson, 2018; Spence et al., 2011; Sweeney et al., 2019). Web-based psychological intervention programs that include guidance, which can be supportive and practical instead of deliberately therapeutic, have been found to have better outcomes compared to treatments with no guidance (Andersson and Cuijpers, 2009; Andersson and Titov, 2014; Richards and Richardson, 2012). A clinician may be involved in web-based psychological intervention programs in various ways and with varying levels of contribution, but it has also been noted that efficient support may come from a sufficiently trained non-clinician (e.g., Lappalainen et al., 2007; Räsänen et al., 2016). Researching the potential in web-based psychological intervention programs may reveal important information on how to reach groups that might have previously been overlooked.

Adherence, compliance, and retention have been an area of interest in medical literature (e.g., see Borus and Laffel, 2010; Dodds et al., 2003; Kyngäs and Rissanen, 2001), and though not completely unknown in other fields, the exact measurements and mechanisms of adherence in the context of web-based psychological intervention programs have yet to be identified (Donkin et al., 2011; Short et al., 2018; Sieverink et al., 2017). Adherence to web-based psychological intervention program was in the present study represented by the frequency of activity shown within the intervention program (Neil et al., 2009; Perski et al., 2017; Ryan et al., 2018). Investigating factors associated with engaging in web-based psychological intervention programs may provide information on how to increase participants' completion of the program, thus increasing the probability of gaining as much as possible from the program.

### 1.1 ACT-based interventions among adolescents

Acceptance and commitment therapy (ACT) is a third-wave cognitive behavioral therapy that is based on functional contextualism and sees psychological events as a set of interactions between whole organisms and different contexts (Hayes et al., 1999). ACT endeavors to identify language processes that produce ineffective strategies of control (Hayes, 2004; Hayes et al., 1999). The aim in ACT is to increase psychological flexibility, which is divided into six key processes: values, committed action, acceptance, contact with the present moment, cognitive defusion, and self as context (Hayes et al., 2006). Different metaphors, exercises, and homework are used to recognize the connection between thoughts, emotions, and behavior.

Research concerning adult populations is relatively more common in ACT-based interventions than among adolescent populations (e.g., see Halliburton and Cooper, 2015; Powers et al., 2009; Ruiz, 2012). Research among adolescents has, however, been conducted on various topics such as depressive symptoms (Ames et al., 2013; Hayes et al., 2011; Lappalainen et al., 2021), ADHD (Zylowska et al., 2008), stress (Livheim et al., 2014; Puolakanaho et al., 2019b), anorexia (Heffner et al., 2002) and anxiety (Biegel et al., 2009). Findings among adult as well as adolescent populations have suggested ACT-based interventions to be effective for a range of mental and physical health problems and for supporting psychological well-being.

A psychological intervention program designed for adolescent populations should account for the individual differences in biological, psychological and social domains to make the program developmentally appropriate. Individual differences have been addressed in ACT-based programs by providing multiple brief, experiential and interactive exercises and by utilizing demonstrative examples, drawing, role-playing, games, writing, art, and other concrete tools (Hayes and Ciarrochi, 2015). These support adolescents to become aware of their inner and outer experiences, to accept them and to detached from them (Hayes et al., 2010; Kingery et al., 2006). Furthermore, motivation for treatment can be strengthened by using an adolescent's own fields of interest in seeking for values and actions based on them. (Halliburton and Cooper, 2015; Kingery et al., 2006; Puolakanaho et al., 2019a; Wicksell et al., 2009). Insights and skills obtained during the intervention are important to transmit from the intervention to the adolescent's everyday life and social relationships, in which friends and peer support can offer an applicable platform for rehearsal (Kingery et al., 2006; Livheim et al., 2014; Puolakanaho et al., 2019b).

### 1.2 Factors anticipating adolescents' engagement with and experiences of web-based ACT interventions

Adherence to treatment by adolescents has been found to be connected to factors such as self-regulation skills (Berg et al., 2014; Graziano et al., 2011; Miller et al., 2020) and the perceived threat of physical disease affecting mental well-being (Kyngäs, 2007). Demographic variables such as age, gender, and parents' educational level have received mixed results, with some studies finding connections to adherence and some not (see, e.g., Calear et al., 2013, Christensen et al., 2009, Garrido et al., 2019, Kristensen et al., 2018, Marko-Holguin et al., 2010, Mattila et al., 2016, Neil et al., 2009, Williams et al., 2006). Higher average school grades have been associated with greater uptake intentions for web-based psychological interventions (Lillevoll et al., 2014). Regarding adolescents' well-being, pre-intervention depression symptom severity has also received mixed results between studies as some have reported higher adherence to associate with higher depression symptom severity but others have reported the associations to occur with lower depression symptom severity (Batterham et al., 2008; Calear et al., 2013; Christensen et al., 2009; Korbel et al., 2007; Kristensen et al., 2018; Neil et al., 2009; Nock and Ferriter, 2005).

Support and encouragement from others have also been identified to play a role in adolescents' help-seeking process and treatment adherence (Gulliver et al., 2010; Kyngäs and Rissanen, 2001; Kyngäs, 2007). Adolescence is a developmentally sensitive period, and the effects of acceptance and rejection in relationships with parents, teachers, and peers during this period hold risks and buffers that contribute to later social and emotional adjustment (Fredriksen and Rhodes, 2004; Kiuru et al., 2019; Parker and Asher, 1987; Sentse et al., 2010; Videon, 2005; Wanders et al., 2020). Due to the involvement of interpersonal relationships in adjustment, behavior, and adherence, there is a possibility that they could also contribute to predicting adolescent usage and the experiences gained from a psychological intervention program.

Adolescent perceptions and attitudes towards web-based psychological intervention programs seem to be generally positive (Sweeney et al., 2019; Van Voorhees et al., 2009; Whittaker et al., 2012). Greater perceived benefits of web-based interventions have been associated with greater liking for technology and more positive attitudes towards mental health, whereas these factors, along with being of female gender, having no prior experience with web-based psychological interventions, and having greater knowledge of them were associated with higher perceived helpfulness (Sweeney et al., 2019). Therapy programs with more surface credibility, that is, looking and feeling competent, have been suggested to result in higher engagement and satisfaction with the program (Wozney et al., 2017). Users are more likely to be motivated to use the program when it presents the concepts in an engaging way, allows interaction, and is relatable and easy to navigate (Wozney et al., 2017). ACT intervention programs in web-based forms (Lappalainen et al., 2015; Lappalainen et al., 2007; Puolakanaho et al., 2019b) and as a web-based adjunctive tool (Levin et al., 2015) have received positive feedback, with participants largely satisfied with the intervention and found it useful in regards to learning mindfulness, acceptance and value-related skills, whose promotion is the central aim in ACT interventions.

Based on previous findings stemming mainly from face-to-face ACT interventions, gender, academic achievement, self-regulation (in terms of effortful control), and psychological well-being (using measurements of pre-intervention stress level, life satisfaction, and depressive symptoms) were selected among the possible antecedents of usage and experiences of a web-based psychological intervention program in the current study. In addition to examining these biological and psychological domains, the individual differences in the social domain were also examined: environmental antecedents of usage and experiences of a web-based ACT program included parents' educational level, peer acceptance, peer rejection, and perceived closeness to and conflict with parents and teachers.

### 1.3 Current study

We examined Finnish adolescents' usage activity and experiences of a brief universal ACT-based web intervention program called Youth Compass. Previously, studies on Youth Compass have shown adolescents to experience positive gains in their psychological well-being (Lappalainen et al., 2021; Puolakanaho et al., 2019b). The current study investigated how individual and environmental antecedents predict adolescents' usage activity and user experiences in the Youth Compass program. Making behavioral changes tends to require frequent practice and ongoing effort to consolidate and integrate them into everyday life. Thus, we determined *usage activity* of the intervention as an indicator of frequent practice. Usage activity refers in the present study to the frequency of accessing the web-based intervention, operationalized as the number of separate days a participant used the intervention program during the five-week intervention period (see also Perski et al., 2017; Ryan et al., 2018). In addition, we were interested in participants' intervention experiences, that is, their perceptions of satisfaction with and the usefulness (i.e., learning of mindfulness, acceptance and value-related skills) of the intervention program. Adolescent intervention experiences and their perceptions of learning well-being skills may reveal valuable information on what could make a program more appealing to adolescents, thus encouraging them to use it more and benefit from it as much as possible.

The more detailed research questions were the following:

1) To what extent are individual (i.e., gender, effortful control, academic achievement, and psychological well-being) and environmental (i.e., parents' level of education and relationships with peers, parents, and teachers) antecedents associated with adolescents' usage activity and their experiences of the web-based Youth Compass intervention program?
2) What subgroups of adolescents can be identified based on their usage activity and experiences (i.e., perceived usefulness and intervention satisfaction) of the web-based Youth Compass intervention program?
3) To what extent are individual and environmental antecedents associated with subgroup membership in regard to adolescents' usage activity and experiences of the web-based Youth Compass intervention program?

## 2 Method

### 2.1 Participants and procedure

Sample selection and randomization to intervention groups was done in two phases. First, a subsample from the community sample of a broader longitudinal project (about 800 participants) was randomized to partake in the universal Youth Compass intervention at fall of 2017, at the beginning of Grade 9. The intervention targeted the whole population of students, i.e. participants were not identified as having mental health problems. All participants and their parents had given written consent to participate in the intervention prior to randomization. Second, the subsample was randomized into two intervention groups: group with online support in addition to face-to-face support (*n* = 83) and group with only online support (*n* = 82). Two participants from each randomized group did not participate in pre-measurement condition at Grade 9 early fall (*n* = 81 and *n* = 80 respectively). In turn, four participants from group of online support in addition to face-to-face support did not take part in post-measurement at Grade 9 late fall (*n* = 77 and *n* = 80 respectively).

Participant characteristics are presented in Table 1. Pre- and post-measurements were carried out during school hours and the adolescents used the intervention program on their leisure time. Each participant was given a personal coach who provided online support and reminders about the program in the form of instant text messages via the WhatsApp application. The coaches were bachelor or master's level psychology students who were trained in the acceptance and commitment therapy approach prior to the intervention. In the present investigation all the adolescents randomized in the Youth Compass intervention (*n* = 161) were analyzed as one group. Because the type of support (only online support or online support in addition to minimal face-to-face support) did not contribute in a statistically significant way to adolescents' usage activity, perceived usefulness and satisfaction with the intervention program (*p* > .05 in all), the type of intervention group was not included in the subsequent analyses of this study.

**Table 1:** Demographic information on Youth Compass intervention participants (*n* = 161).

Characteristic	All Youth Compass participants
Age: *M* (*SD*)	15.26 (0.32)
Gender: Female, *n* (%)	81 (50)
Mother tongue	
Finnish *n* (%)	151 (94)
Other than Finnish *n* (%)	6 (4)
Bilingual *n* (%)	3 (2)
Lives with	
Mother and father *n* (%)	111 (71)
With mother or father *n* (%)	16 (10)
Alternately with mother and father *n* (%)	23 (15)
Other^a^*n* (%)	7 (4)
Mother's education level^b^*M* (*SD*)	4.32 (1.33)
Father's education level^b^*M* (*SD*)	3.87 (1.50)

*Note*. *M* = mean, *SD* = standard deviation.

a Participant lives with mother and stepfather, father and stepmother, in foster care, or in approved home.

b Education level on scale of 1 to 7, where 1 = no vocational training; 7 = postgraduate degree, i.e., licentiate, doctorate.

During the five-week Youth Compass intervention, each week presented a different module focusing on a specific ACT-based theme: (1) finding personal interests, (2) awareness of self, skills of acceptance and cognitive defusion, (3) being in the present, (4) self as context and self-compassion, and (5) applying important actions to social life and compassion towards others. A single module consisted of an introduction and three different levels in which at least two exercises were to be completed to advance in the program. The participant needed to complete at least six separate exercises that were approximately five minutes long in order to finish the module. For further details about the intervention program, see Puolakanaho et al. (2019b) and Lappalainen et al. (2021).

Individual and environmental antecedents were measured in the pre-measurement carried out before the intervention, in the early fall of Grade 9. Adolescents' activity in using the intervention program was measured during the intervention, and adolescents' experiences of the intervention were measured in the post-measurement carried out after the intervention, in the late fall of Grade 9.

### 2.2 Outcome measures

#### 2.2.1 Intervention-related usage activity and experiences of the intervention (Grade 9, late fall, after the five-week Youth Compass intervention)

**Usage activity of the web-based intervention program**

Adolescents' usage activity was measured by the number of separate days a participant used the intervention program. In other words, higher number of separate usage days during the five-week intervention period was considered to represent higher usage activity.

**Satisfaction with the intervention program**

Adolescents were asked to evaluate two questions on a scale from 4 to 10 (4 = very unsatisfied; 10 = very satisfied) how satisfied were they with the Youth Compass experience generally and how satisfied were they with the Youth Compass program. Mean scores were calculated to measure the level of satisfaction with the intervention program (*α* = .95).

**Perceived usefulness of the intervention program**

Adolescents reported their perceived usefulness of the intervention program in regards to learning mindfulness, acceptance and value-related skills, the promotion of which is in the heart of ACT interventions (Flaxman et al., 2013; Hayes et al., 1999; Twohig et al., 2010). Adolescents answered to seven statements on a scale from 4 to 10 (4 = I haven't learned at all; 10 = I have learned very much) relating to skills learned with the help of the program. These included, for example, “I have learned to notice my thoughts, emotions, and feelings better”; “I have learned to distance myself from thoughts and emotions.” Mean scores across these ratings were calculated to measure how useful the intervention program was perceived to be (*α* = .96).

#### 2.2.2 Individual-related antecedents (Grade 9, early fall, before the Youth Compass intervention)

**Gender**

Adolescents' gender was coded as 0 = female and 1 = male.

**Temperamental effortful control**

Temperamental effortful control refers to the capacity in stimuli response and attentional regulation (Eisenberg, 2012; Rothbart and Jones, 1998; Rothbart, 2011), and is used in this study as a measure for self-regulation. Using the short version of the revised Early Adolescent Temperament Questionnaire (EATQ–R; Capaldi and Rothbart, 1992, Ellis and Rothbart, 2001, Ellis, 2002, short self-report version translated to Finnish by Katri Räikkönen-Talvitie and the Developmental Psychology Research Group of University of Helsinki), the adolescents assessed their temperament and self-regulation on a scale from 1 to 5 (1 = almost never true; 5 = almost always true). The subscale for temperamental effortful control consists of seven statements, such as “It is easy for me to really concentrate on homework problems”; “I have a hard time finishing things on time” (reversed). A mean score for individual adolescents' effortful control (*α* = .80) was calculated. Higher temperamental effortful control is indicated by a higher mean score.

**Academic achievement**

Adolescents provided information on their overall academic achievement as a self-evaluated grade point average. The grade range in the Finnish school system is from 4 to 10, where 5 is the lowest and 10 the highest accepted grade. Self-reported school grades have been shown to have a correlation of .86 with the actual grades from school registers (Sainio et al., 2019).

**Stress**

After explaining stress in written form (“Stress refers to a situation where people feel tensed, restless, nervous, or anxious and have difficulties sleeping due to the things wandering in their mind”), the participants answered the question “Do you feel this kind of stress at the moment?” using a six-point scale (1 = not at all; 6 = very much). The single-item measure for stress covers psychological symptoms and sleep disturbances, which are central indicators of stress (Elo et al., 2003). According to Elo et al. (2003), the validity of the single-item measure for stress is supported by its congruence with other validated measurements for mental well-being like the General Health Questionnaire (GHQ) and 36-Item Short Form Survey (SF–36).

**Depressive symptoms**

Adolescents were asked to assess their mood during the last month by completing the Depression Scale (DEPS; Salokangas et al., 1995, see also Kiuru et al., 2012, Poutanen et al., 2010). The questionnaire consists of 10 items (e.g., “I feel sad”; “I feel that my future is hopeless”), which are answered on a scale from 0 to 3 (0 = not at all; 3 = very much), making the range of the sum score 0–30 (*α* = .95). A higher score refers to a greater severity of depressive symptoms. A score of 9–10 is considered as the cut-off for some depressive symptoms and a score of 11–12 as the clinical cut-off (Poutanen et al., 2010).

**Life satisfaction**

To measure adolescents' life satisfaction, the Finnish version of the Satisfaction with Life Scale (SWLS; Diener et al., 1985, see also Mauno et al., 2018) was used. The scale consists of five items (e.g., “I am satisfied with my life”; “So far I have gotten the important things I want in life”) which are answered on a scale of 1 to 5 (1 = completely disagree; 5 = completely agree). Mean scores were then calculated from the items, so the range of the life satisfaction scale was between 1 and 5 (*α* = .92), where a higher score indicated higher life satisfaction.

#### 2.2.3 Environment-related antecedents (Grade 9, early fall, before the Youth Compass intervention), that is, the level of parental education and quality of relationships with peers, parents, and teachers were measured in the fall of Grade 9 before the Youth Compass intervention

**Level of parents' education**

Adolescents' parents reported their level of education with a corresponding value of 1 to 7 (1 = no vocational training; 7 = postgraduate degree, i.e., licentiate, doctorate).

**Peer acceptance and peer rejection**

Adolescents' peer acceptance and peer rejection in Grade 9 was measured using a sociometric nomination procedure. Adolescents nominated up to six peers in the same grade but outside of their own class with whom they most like to spend time (positive nominations) and up to six peers in the same grade but outside of their own class with whom they least like to spend time (negative nominations) during schooldays. Peer acceptance represents the number of positive nominations each adolescent received, standardized within grade level, whereas peer rejection represents the number of negative nominations each adolescent received, standardized within grade level. Sociometric nominations provide valid, stable, and reliable assessments of peer acceptance during childhood and adolescence (Bukowski et al., 2012).

**Closeness to and conflict with parents**

To measure the adolescents' relationship with their parents, they rated their experiences using the Child–Parent Relationship Scale (CPRS; Driscoll and Pianta, 2011, see also Mauno et al., 2018). The questionnaire measures experienced closeness with five items (e.g., “I have a close and warm relationship with my mother/father”) and conflict with six items (e.g., “I often argue with my mother/father”), which are answered on a scale from 1 to 5 (1 = not true at all; 5 = completely true). Mean scores were calculated to measure the adolescents' perceived closeness to and conflict with their mothers (*α* = .89, *α* = .87 respectively) and fathers (*α* = .90, *α* = .87 respectively).

**Closeness to and conflict with teachers**

To measure the adolescents' relationship with their teacher they rated their experiences using the Student–Teacher Relationship Scale (STRS–Short Form; Pianta, 2001). On a scale from 1 to 5 (1 = not true at all; 5 = completely true), adolescents answered five items regarding closeness (e.g., “I have a close and warm relationship with my teacher”) and six items regarding conflict (e.g., “I often argue with my teacher”). The mean scores were calculated across these ratings to estimate the adolescents' perceptions of their closeness (*α* = .77) and conflict (*α* = .88) with their teacher.

### 2.3 Analysis strategy

The first aim of the current study was to examine the extent to which adolescent- and environment-related factors are related to adolescents' usage activity and experiences of the Youth Compass intervention program. This research question was answered with variable-oriented analysis methods, that is, correlations and regression analyses. Further aims of the study were to identify subgroups of adolescents based on their usage activity and experiences with Youth Compass and explore associations of individual and environment-related factors for membership in these subgroups. These research questions were answered by person-oriented analysis methods using latent profile analysis (i.e., LPA, mixture modelling, Muthén and Asparouhov, 2006, Vermunt and Magidson, 2002). The analysis seeks to identify the smallest number of latent groups that adequately describe the mean profiles of observed continuous variables as well as to enable investigation of the correlates of these subgroups. All of the variables (i.e., usage activity, intervention satisfaction and perceived usefulness of intervention) were standardized before the LPA analyses.

The latent profile analyses were conducted using Mplus (version 8.4, Muthén and Muthén, 1998–2017). The following indices were used to select the number of latent groups from the latent profile analyses: (a) the fit of the model, (b) the average latent class probabilities and the number of adolescents to be located in a latent group, and (c) the practical usefulness, theoretical justification, and interpretability of the latent group solution (Bauer and Curran, 2003; Muthén, 2003). The fit of the model was evaluated by the following criteria: (a) the Bayesian information criterion (BIC), (b) the Lo–Mendell–Rubin adjusted likelihood ratio test (aLRT), (c) the Vuong–Lo–Mendell–Rubin likelihood ratio test (VLMR), and (d) Bootstrap Likelihood Ratio Test (BLRT; McLachlan and Peel, 2000). Lower values of information criteria indicate a better model, and significant aLRT and VLMR test results indicate a higher number of groups. Finally, comparison between latent groups in regard to individual and environment-related antecedents was conducted using the Mplus auxiliary function with the BCH method (Asparouhov and Muthén, 2014a, Asparouhov and Muthén, 2014b). Using the auxiliary function provides an opportunity to investigate differences between latent groups without impacting the final latent group solution. The group comparison was based on a Wald Chi-square test of statistical significance.

## 3 Results

Individual- and environment-related antecedents were measured in the early fall of Grade 9, and adolescents' usage activity and experiences in late fall after the intervention. Table 2 presents the means, standard deviations, and ranges of the observed variables. The amount of days participants used the program varied between a minimum of 1 and maximum of 20 days, with a mean of 5.9 days. Ninety percent of the participants used the intervention on 1 to 9 separate days during the intervention program.

**Table 2:** Descriptive statistics of the observed variables (*n* = 161).

Variable	*M*	*SD*	Range
Individual-related antecedents			
Gender (% of males)	0.50	0.50	1.00
Effortful control	3.28	0.70	3.57
Academic achievement (self-reported GPA)	7.89	0.92	4.50
Stress	2.95	1.45	5.00
Depressive symptoms	7.08	7.38	29.00
Life satisfaction	3.54	0.93	4.00

Environment-related antecedents			
Mother's level of education	4.32	1.33	6.00
Father's level of education	3.87	1.50	6.00
Peer acceptance	4.56	2.69	13.00
Peer rejection	2.52	2.75	12.00
Perceived closeness with mother	3.66	1.04	4.00
Perceived closeness with father	3.26	1.10	4.00
Perceived closeness with teacher	1.85	0.70	3.00
Perceived conflict with mother	2.08	0.92	4.00
Perceived conflict with father	1.92	0.87	3.67
Perceived conflict with teacher	1.86	0.91	4.00

Adolescents usage activity and experiences of the intervention			
Usage activity (sum of days)	5.90	3.29	19.00
Perceived usefulness (scale 4–10)	6.86	1.50	5.43
Intervention satisfaction (scale 4–10)	7.45	1.42	6.00

*Note*. *M* = mean, *SD* = standard deviation. Gender coded as 0 = female, 1 = male.

### 3.1 Variable-oriented results: correlations and regression analyses

Our first research question examined the extent to which individual and environmental antecedents were associated with adolescents' usage activity and their experiences of the web-based Youth Compass intervention program. The correlations regarding individual-related antecedents, presented in Table 3, showed that compared to male adolescents, female adolescents had higher usage activity, were more satisfied with the intervention and were more likely to perceive the intervention as useful. Higher temperamental effortful control was related to higher usage and perceived usefulness. Higher academic achievement was related to higher usage activity and intervention satisfaction. Adolescents' stress before the intervention was connected with usage activity as such that adolescents experiencing higher stress used the program more often during the intervention than their less stressed counterparts did. In turn, life satisfaction and depressive symptoms before the intervention were not related to adolescents' usage activity or experiences of the intervention.

**Table 3:** Correlations between individual- and environment-related factors and adolescents' usage activity and experiences of the Youth Compass intervention (*n* = 161).

Variable	Usage activity	Perceived usefulness	Intervention satisfaction
Individual-related factors			
Gender	−.27⁎⁎	−.16⁎	−.25⁎⁎
Temperamental effortful control	.16⁎	.23⁎⁎	.14
Academic achievement (self-reported GPA)	.27⁎⁎	.12	.18⁎
Stress	.18⁎	.03	.13
Depressive symptoms	.09	−.06	.08
Life satisfaction	−.06	.08	−.10

Environment-related factors			
Mothers' level of education	.05	.07	.06
Fathers' level of education	−.08	.02	.08
Peer acceptance	.13	.24⁎⁎	.20⁎
Peer rejection	−.08	−.07	−.09
Perceived closeness with mother	.02	.27⁎⁎	.19⁎
Perceived closeness with father	.08	.23⁎⁎	.06
Perceived closeness with teacher	.07	.27⁎⁎	.13
Perceived conflict with mother	−.02	−.08	−.10
Perceived conflict with father	−.06	−.03	−.08
Perceived conflict with teacher	−.27⁎⁎	−.18⁎	−.28⁎⁎

*Note.* Gender 0 = female, 1 = male.

⁎ *p* < .05.

⁎⁎ *p* < .01.

The correlations regarding environmental factors showed that higher peer acceptance was related to higher intervention satisfaction and perceived usefulness. Being rejected by one's peers, on the other hand, was not found to be connected to usage activity or experiences of the program. Adolescents experiencing a closer relationship with their mother reported being more satisfied with the intervention and had higher levels of perceived usefulness. An adolescent's closer relationships with their father and teachers were found to be related to higher levels of perceived usefulness. Activity, perceived usefulness, and satisfaction were all found to correlate with the adolescents' experiences of conflict with their teacher. That is, adolescents reporting higher levels of conflict with their teachers were more likely to be less active in using the intervention program, less satisfied with it, and to perceive it as less useful. Perceived conflict with parents, however, had no connection to usage activity or experiences of the intervention. Parents' level of education was also found to be unrelated to usage activity and experiences of the intervention.

Next, we carried out regression analyses to predict adolescents' usage activity and experiences of the Youth Compass intervention by individual- and environment-related antecedent factors. When antecedent factors were controlled for each other it was found that only the effects of gender (usage activity *β* = −.20, *SE* = .67, *p* = .05; perceived usefulness *β* = −.29, *SE* = .30, *p* = .005; intervention satisfaction *β* = −.26, *SE* = .29, *p* = .01) and temperamental effortful control (usage activity *β* = .22, *SE* = .49, *p* = .05; perceived usefulness *β* = .38, *SE* = .22, *p* = .001; intervention satisfaction *β* = .28, *SE* = .21, *p* = .01) were significant. The models' *R*^2^ values for usage activity, perceived usefulness, and intervention satisfaction were .15, .19, and .16 respectively.

### 3.2 Person-oriented analyses: subgroups of adolescents in regard to their usage activity and experiences of Youth Compass

Our second aim was to examine what subgroups of adolescents can be identified based on their usage activity, perceived usefulness and satisfaction with the Youth Compass intervention program. A series of latent profile analyses (LPA) was conducted to identify subgroups and estimate the parameters for them. The fit of the model was evaluated by criteria based on BIC, aLRT, VLMR, and BLRT. Lower values of information criteria indicate a better model, and significant test results indicate a higher number of groups. Table 4 shows the LPA fit indices and class frequencies with different numbers of profiles.

**Table 4:** Fit indices and class frequencies for latent profile analyses with different numbers of latent profiles (*n* = 157).

No. of groups	BIC	*p* value of LMR	*p* value of VLMR	*p* value of BLRT
1 (*N* = 157)	1304.34			
2 (*n1* = 48, *n2* = 109)	1156.75	<.001	<.001	<.001
3 (*n1* = 78, *n2* = 30, *n3* = 49)	1140.36	.060	.064	<.001
**4 (*n1* = 65, *n2* = 29, *n3* = 12, *n4* = 51)**	**1136.53**	**.020**	**.022**	**.05**
5 (*n1* = 50, *n2* = 64, *n3* = 29, *n4* = 11, *n5* = 3)	1152.27	.333	.340	1.00

*Note.* BIC = Bayesian Information Criterion; LMR = Lo–Mendell–Rubin Adjusted Likelihood Ratio Test; VLMR = Vuong–Lo–Mendell–Rubin Likelihood Ratio Test; BLRT = Bootstrapped Likelihood Ratio Test.

**Fig. 1:**
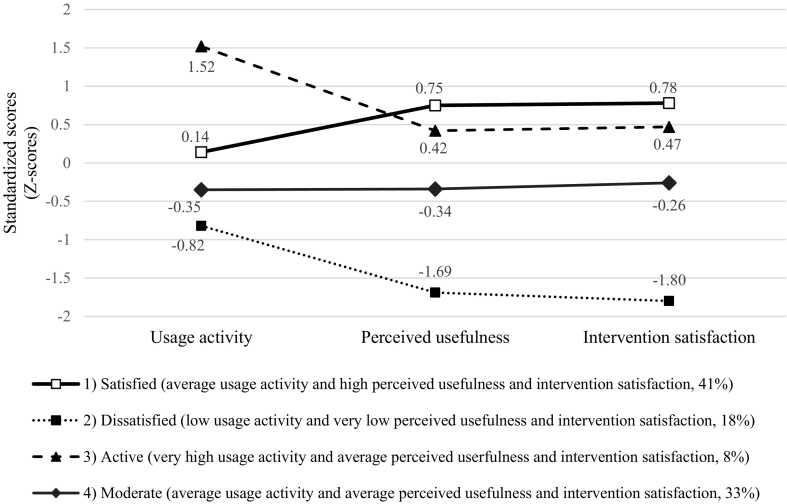
Latent mean profiles of adolescents' usage activity and experiences of Youth Compass.

Based on the fit indices, the four-group solution was concluded to fit the data best. Fig. 1 depicts the latent mean profiles of adolescents' usage activity, perceived usefulness, and satisfaction in Youth Compass intervention program.

According to their mean score profiles (Fig. 1), these groups were labelled: (1) ‘satisfied’ (average usage activity and high perceived usefulness and intervention satisfaction, 41% of participants), (2) ‘dissatisfied’ (low usage activity and very low perceived usefulness and intervention satisfaction, 18% of participants), (3) ‘active’ (very high usage activity and average perceived usefulness and intervention satisfaction, 8% of participants), and (4) ‘moderate’ (average usage activity and average perceived usefulness and intervention satisfaction, 33% of participants).

### 3.3 Person-oriented analyses: antecedents' associations with subgroup membership

Our final aim was to explore the associations of individual and environment-related factors with membership in subgroups regarding usage activity and experiences of the Youth Compass intervention program. The estimated means and standard errors and results for comparisons between the latent profiles in regard to individual and environmental factors are shown in Table 5.

**Table 5:** Estimated means and standard errors and results for the comparisons between the latent profiles in regard to individual and environmental factors.

	1) Satisfied = Average activity and high perceived usefulness and intervention satisfaction (*n* = 65)		2) Dissatisfied = Low activity and very low perceived usefulness and intervention satisfaction (*n* = 29)		3) Active = Very high activity and average perceived usefulness and intervention satisfaction (*n* = 12)		4) Moderate = Average activity and average perceived usefulness and intervention satisfaction (*n* = 51)		*χ2(3)*	*p*
Variable	*M*	*SE*	*M*	*SE*	*M*	*SE*	*M*	*SE*		
% of males	0.43	0.07	0.80	0.09	0.23	0.14	0.50	0.08	16.34	.001
Effortful control	3.48^b^	0.10	3.13^a^	0.14	3.24^ab^	0.17	3.16^ab^	0.12	5.78	.123
Academic achievement	8.06^ab^	0.11	7.63^b^	0.23	8.42^a^	0.24	7.67^b^	0.17	10.15	.017
Stress	2.81^ab^	0.21	2.46^a^	0.29	3.61^b^	0.43	3.15^ab^	0.25	5.61	.132
Depressive symptoms	7.00	1.14	4.73	1.24	9.48	2.76	7.61	1.22	4.29	.232
Life satisfaction	3.57	0.13	3.83	0.18	3.31	0.29	3.44	0.17	3.41	.333
Level of parental education	4.46	0.20	4.59	0.266	4.18	0.60	4.13	0.25	1.66	.647
Peer acceptance	0.17^a^	0.14	−0.38^b^	0.18	0.08^ab^	0.28	−0.06^ab^	0.14	6.38	.094
Peer rejection	0.17	0.16	0.20	0.22	0.01	0.27	0.18	0.16	0.33	.95
Closeness with mothers	3.96^a^	0.14	3.07^b^	0.22	3.33^ab^	0.37	3.75^a^	0.17	11.83	.008
Conflict with mothers	1.92	0.13	2.19	0.23	2.34	0.28	2.16	0.16	2.40	.494
Closeness with fathers	3.45^a^	0.17	2.83^b^	0.20	3.05^ab^	0.38	3.31^ab^	0.18	6.26	.099
Conflict with fathers	1.83	0.12	2.05	0.22	2.06	0.27	1.92	0.14	1.04	.793
Closeness with teachers	2.02^a^	0.10	1.53^b^	0.11	1.87^ab^	0.31	1.84^ab^	0.12	11.407	.010
Conflict with teachers	1.73^a^	0.11	2.25^b^	0.23	1.23^c^	0.11	1.97^ab^	0.16	25.60	<.001

*Note. M* = mean, *SE* = standard error. Means or proportions within a row sharing the superscripts (a through c) are pairwise significantly different at the level of *p* < .05.

There were relatively more male adolescents in the dissatisfied profile than in the other three profiles. The academic achievement of adolescents in the active profile was found to be higher compared to the dissatisfied and moderate profiles. No significant differences were found between profiles in regard to depressive symptoms or life satisfaction. In temperamental effortful control and stress, mean test scores across all the means were not significant, and only significant pairwise differences were found: temperamental effortful control was lower in the dissatisfied profile compared to the satisfied profile, and adolescents belonging to the active profile reported more stress than did those in the dissatisfied profile.

Adolescents in the satisfied and moderate profiles were found to have a closer relationship with their mother compared to adolescents in the dissatisfied profile. Adolescents in the dissatisfied profile had lower closeness to their teacher compared to those in the satisfied profile. Adolescent–teacher conflict was lower in the active profile than it was in the other three profiles. In addition, the perceived conflict between adolescent and teacher was found to be higher in the dissatisfied profile than in the satisfied profile. No differences were found between profiles in parents' education, peer rejection, or conflicts with parents. In peer acceptance and closeness with fathers, mean test scores across all the means were not significant, and only significant pairwise differences were found: peer acceptance was found to be lower among adolescents in the dissatisfied profile compared to those in the satisfied profile, and satisfied profile adolescents had a closer relationship with their father compared to those in the dissatisfied profile.

## 4 Discussion

This study examined the usage activity, perceived usefulness and satisfaction of 15-year-old Finnish adolescents regarding Youth Compass, a five-week universal web-based acceptance and commitment therapy intervention program. Pre- and post-measurements were carried out during school hours and the adolescents used the intervention program on their own leisure time. In the current study, 82% of adolescents were moderately to highly active in using Youth Compass and rated their user experiences as average to high in terms of perceived usefulness and satisfaction with the program. The generally positive stance seems to be in line with findings from other studies on adolescents' views on web-based psychological intervention programs (Sweeney et al., 2019; Van Voorhees et al., 2009; Whittaker et al., 2012).

Regarding the first research question, the results of the regression analysis showed that especially adolescent gender and self-regulation were related to adolescents' usage activity, perceived usefulness and satisfaction (*R*^*2*^ = .15, .19, and .16 respectively) with Youth Compass. In previous studies, the findings on gender have been mixed, but in those observing a connection between gender and adherence or perceptions of web-based psychological interventions (e.g., Garrido et al., 2019; Neil et al., 2009; Sweeney et al., 2019), the results have pointed in a similar direction as in this study. Female adolescents were found in the present study to be more active, have higher rates of satisfaction, and be more likely to perceive the intervention as useful compared to male adolescents. This could be seen as reflecting gender-based differences in receptiveness to health intervention strategies (Orji, 2014), suggesting that female adolescents may be more active Youth Compass users because the program is more appealing to them. Consequently, this highlights the need for web-based psychological intervention programs to better account also for the interests of male adolescents and find appropriate motivation strategies (Garrido et al., 2019; Puolakanaho et al., 2019b). Measured in the present study in terms of temperamental effortful control, self-regulation showed associations with usage activity and perceived usefulness of the intervention. This finding supports previous results on self-regulation's role in achieving health-related outcomes (Berg et al., 2014; Miller et al., 2020), and implies that the Youth Compass program could potentially be more beneficial to adolescents with higher self-regulation skills because they are more likely to engage with the program material and its objectives. Higher perceived usefulness of the Youth Compass program could possibly be due to higher adherence facilitated by self-regulatory mechanisms. This raises the question of how a web-based psychological intervention program can respond to individually varying skill sets such as those relating to attention regulation or comprehension. To support this individualization, tailoring the program, for example, to better address users' personal needs has been suggested (Morrison et al., 2014; Neil et al., 2009).

Other associations between usage activity and intervention experiences were found with academic achievement, stress, peer acceptance, conflict with teacher, and closeness to parents and teacher. These, however, did not show significant predictability in regression analysis. No associations were found between intervention satisfaction or perceived usefulness and depressive symptoms, life satisfaction, parents' educational level, peer rejection, or perceived conflict with parents. Academic achievement's relation to usage and intervention experiences could be explained by the previously found higher uptake intentions (Lillevoll et al., 2014) and the tendency of students with higher average grades to participate more in extracurricular activities (Zaff et al., 2003). Close relationships to adults and peer acceptance were associated with intervention usage and experiences, whereas peer rejection and conflict, apart from conflict with teacher, were not. The result seems to support the view that positive interpersonal relationships may contribute to adolescent adherence (Gulliver et al., 2010; Kyngäs, 2007). In terms of rejection and conflict, teacher–student conflict was the only one to show associations with usage activity and intervention experiences. It is possible that teacher–student dynamics are more highlighted in the results if the intervention program has been associated by the participants with school-related work because pre- and post-measurements were made during schooldays. Parents' educational level, here reflecting socioeconomic status, was not associated with intervention usage or experiences, which is different to what has been suggested in previous studies on treatment adherence (see Kristensen et al., 2018; Nock and Ferriter, 2005). This difference could be explained by the fact that access to technology and mobile devices has rapidly grown and is not necessarily limited by socioeconomic status. Access to technology, consequently, allows access to treatment regardless of location or geographical distances.

In regards to psychological well-being, life satisfaction and the severity of depression symptoms were not found to be connected to usage activity or intervention experiences. Previous findings on adherence have different views on the relationship between pre-intervention depression severity and adherence (Batterham et al., 2008; Calear et al., 2013; Christensen et al., 2009; Korbel et al., 2007; Kristensen et al., 2018; Neil et al., 2009; Nock and Ferriter, 2005), but the current study differs from these by finding no association. One explanation for this could be that the Youth Compass program's purpose is to promote psychological well-being as a universal intervention among a community sample of adolescents, whereas previous research has mostly addressed web-based psychological intervention programs among clinical samples with specific diagnoses or clinically significant symptom levels. Results on life satisfaction and depression symptoms in relation to usage activity and intervention experiences could indicate that adolescents with varying levels of life satisfaction and depression symptoms would be as likely to commit to the intervention and to have positive intervention experiences. On the other hand, the present study found higher stress to associate with higher usage activity. In turn, Puolakanaho et al. (2019b) found adolescents with higher stress to experience greater positive gains from the Youth Compass intervention program. These results together suggest that adolescents who have higher distress may use the program more actively. An alternative explanation would be that initially stressed adolescents have stress also about the usage of the program that is shown as performance-orientation towards completing intervention exercises. In future studies it would be important to assess also the orientation how adolescents do the exercises, e.g. their attention, interest and affect (Perski et al., 2017; Short et al., 2018), in addition to usage activity.

Concerning our second aim, a person-oriented approach was used to describe adolescents' usage activity, perceived usefulness and satisfaction with Youth Compass. A series of latent profile analyses showed the solution of four subgroups to fit the data best. The subgroups were labelled ‘satisfied,’ ‘dissatisfied,’ ‘active,’ and ‘moderate’ according to their usage activity and intervention experience mean score profiles. Satisfied profile adolescents (41% of the participants) had an average level of usage activity and high perceived usefulness and intervention satisfaction. Dissatisfied profile adolescents (18% of the participants) had low usage activity and very low perceived usefulness and satisfaction rates. Active profile adolescents (8% of the participants) had very high usage activity and average rates in perceived intervention usefulness and satisfaction. Moderate profile adolescents (33% of the participants) had average rates on usage activity, perceived usefulness, and satisfaction.

Our third aim was to explore the associations of individual and environmental factors with subgroup membership regarding user activity and experiences of Youth Compass. The results showed gender, academic achievement, closeness to mother and teacher, and conflict with teacher to be meaningfully related to subgroup membership. There were more male adolescents in the dissatisfied profile compared to other profiles, which can be seen as reflecting the aforementioned gender-based differences, suggesting that Youth Compass does not appeal to male adolescents to the same extent it does to female adolescents. Adolescents with higher academic achievement were more likely to belong to the active profile. Despite active profile adolescents having high activity rates, they did not seem to show higher perceived usefulness or satisfaction rates compared to adolescents in the moderate profile. In addition, satisfied profile adolescents had higher ratings of perceived usefulness and satisfaction compared to active profile adolescents. Adolescents with low usage activity and very low perceived usefulness and satisfaction rates were associated with lower experienced closeness to their mothers compared to users with average activity and average or high perceived usefulness and satisfaction rates. The contrast between satisfied and dissatisfied profiles showed the biggest difference in terms of closeness to teachers in relation to perceived usefulness and satisfaction. In addition, higher experienced conflict with teachers was found in the dissatisfied profile compared to the satisfied profile. Adolescents of the active profile had, however, the lowest experienced conflict with teachers. One explanation for these differences could be that the intervention program material and exercises may be interpreted as schoolwork or the coaches' involvement as instructing similar to teaching situations. Associating the program with schoolwork may lead to usage that does not emerge based on a desire to affect health-related behavior. This could explain dissatisfied profile adolescents' low engagement and active profile adolescents' high activity without higher perceived usefulness or satisfaction to the intervention program. Despite no significant differences between groups, mean scores for stress, depression, and life satisfaction seemed to suggest that adolescents in the dissatisfied profile showed lower stress and depression and higher life satisfaction compared to other groups. This could be regarded as an implication that dissatisfied profile adolescents did not feel like they needed support for well-being. Alternatively, it could also reflect gender-based differences in prevalence of adolescent ill-being (Aalto-Setälä, 2010; WHO, 2018).

### 4.1 Limitations

The present study also has its limitations. Research in web-based psychological intervention programs requires a more robust consensus in measuring adherence and engagement (Donkin et al., 2011; Short et al., 2018; Sieverink et al., 2017). Different interpretations of what kind of program use can be seen as being more engaged or active may produce different results on what factors can predict it. This study used the concept of usage activity, measured by the amount of separate days an adolescent used the program. Usage activity is a complex entity and using only one measure to represent participants' program usage should be acknowledged as a possible limitation to the interpretation of results. In the future, usage activity or engagement could be determined based on a composition of measurements of, for example, time, activity, and task or module completion (Donkin et al., 2011; Short et al., 2018). The present study aimed to examine individual and environmental antecedents of the usage activity, perceived usefulness (i.e., learning of mindfulness, acceptance and value-related skills), and program satisfaction of adolescents participating in Youth Compass intervention program. Future studies should also address the possible moderating role of usage activity and intervention experiences in actual intervention efficacy. In the present study, the closest proxy to treatment outcome was to assess adolescent perceptions regarding learning mindfulness, acceptance and value-related skills, whose promotion is essential in ACT (Flaxman et al., 2013; Hayes et al., 1999; Twohig et al., 2010).

In addition, the present study lacks a deeper understanding of the adolescents' perspective on the Youth Compass program, such as surface credibility, which has been noted to affect adolescents' engagement and satisfaction with the program (Wozney et al., 2017). More information about this could be obtained by further inquiries about, for example, whether adolescents found the program easy to use or relatable. In generalizing these findings to other online interventions, it should be noted that the measures related to perceived usefulness and satisfaction were specific to the Youth Compass program. Furthermore, the adolescents' attitudes, beliefs and knowledge regarding web-based psychological intervention programs and mental health issues have not been discussed in the present study. Adolescents with lower knowledge of mental health problems have been found to hold more negative attitudes towards them (Sheffield et al., 2004). Beliefs, attitudes, and knowledge of web-based psychological intervention programs have been associated with intervention adherence (Marko-Holguin et al., 2010; Sweeney et al., 2019). Addressing beliefs, knowledge, and attitudes concerning mental health issues and web-based psychological interventions in research could provide important information on how universal interventions could be made more appealing for larger populations of adolescents to enhance their motivation to adhere to web-based psychological intervention programs. The conclusions in the current study are based on associations, and care must be taken to draw definite interpretations.

### 4.2 Conclusions

The results of the present study suggested the presence of different individual and environmental factors that may affect adolescent adherence (reflected in the present study by usage activity) to a web-based universal psychological intervention program and adolescent intervention experiences (reflected in the present study by ratings of perceived usefulness and program satisfaction). Moderate to high usage activity and/or intervention experience rates in the Youth Compass program seemed to be characterized by adolescents' female gender, higher self-regulation skills, good academic performance and positive relationships with adults near them. The results implied that adolescents with varying levels of life satisfaction and depression symptoms would be as likely to commit to the Youth Compass program, but adolescents with higher levels of stress would be more likely to commit to the program compared to their less stressed counterparts. On one hand, it seems likely that adolescents experiencing higher stress may be more motivated to seek support for their well-being. On the other hand, committing to the web-based psychological intervention program seems as likely for adolescents of varying levels of psychological symptoms.

In the future, web-based psychological intervention programs could take individually varying needs and adolescent gender into closer consideration (Garrido et al., 2019; Morrison et al., 2014; Neil et al., 2009). The roles of psychological symptom levels in predicting adolescent adherence and intervention experiences should be further investigated in future studies. . In addition, more research with varying age groups and clinical samples are required to extend knowledge concerning feasibility of the Youth Compass program. The results of the present study contribute to understanding what should be taken into account in making a web-based psychological intervention program appealing to as large population of adolescents as possible.

## Ethical approval

This study was conducted in compliance with APA ethical standards and with the Code of Ethics of the World Medical Association (Declaration of Helsinki) for experiments involving humans. The study was approved by the Ethical Committee of the University of Jyväskylä and has been registered at ClinicalTrials.gov.

## Informed consent

Informed consent was obtained from all the participants of the study.

## Funding

This study was funded by the Finnish Cultural Foundation and the Academy of Finland (No.324638).

## Data sharing and declaration

The datasets generated and/or analyzed during the current study are not publicly available but are available from the corresponding author on reasonable request.

## Declaration of competing interest

The authors declare that they have no conflict of interest. The authors have no financial relationship to the program under examination.
